# Chemical Analysis of Hematite Ore Collected from Pokhari, Nawalparasi, Nepal

**DOI:** 10.1155/2022/4943823

**Published:** 2022-08-31

**Authors:** Ram Bahadur Gharti, Hari Bhakta Oli, Deval Prasad Bhattarai

**Affiliations:** ^1^Department of Mines and Geology, Ministry of Industry, Commerce and Suppliers, Kathmandu, Nepal; ^2^Department of Chemistry, Amrit Campus, Tribhuvan University, Kathmandu, Nepal

## Abstract

Iron is the principal raw material for steel industries and Hematite is a principal ore of iron. Quantitative and qualitative estimation of iron in its ores is a crucial factor before its extraction. In this work, quantitative chemical analysis of iron was carried out from the collected seventy-two hematite samples from Pokhari, Nawalparasi. Sampling of the hematite ore was performed by channel sampling method. Chemical analysis was carried out by gravimetric, titrimetric, UV-Vis spectrophotometric and atomic absorption spectroscopic methods. The findings of different parameters in percentage areas follows: loss on ignition (1.76 ± 0.17), silica (47.06 ± 4.01), and iron (36.75 ± 2.50) by titrimetric analysis. Based on the chemical analysis, the Fe content in the collected hematite sample is in intermediate range. Thus, for the profitable iron extraction, other factors such as coverage of ores, extraction costs, and market value should be considered.

## 1. Introduction

Determination of iron content in rocks and minerals is an important aspect in metallurgical, geochemical, and petrochemical investigations [1]. Iron is the least expensive and most widely used metal which is extensively used for the infrastructure development works, heavy machinery tools, agricultural tools, and household utensils. Hematite and magnetite are commonly exploited iron-bearing ores among the various minerals of iron like hematite (*α*-Fe_2_O_3_ or *α*-polymorph of ferric oxide), maghemite (*γ*-Fe_2_O_3_), magnetite (Fe_3_O_4_), limonite (FeO (OH). nH_2_O or hydrated ferric oxide hydroxide), goethite (*α*-polymorph of limonite), siderite (FeCO_3_), and pyrite (FeS_2_) [2, 3]. Except hematite, other ores are less stable in ambient conditions, and also, contain less amount of iron. The presence of impurities lowers the percentage of iron in the ore. The quality of iron ore is evaluated based on the iron content. More specifically, the ores having iron content above 65% are regarded as high-grade; iron content in the range of 62–64% is medium (average) grade, and iron content below 58% is considered as low-grade ores [4, 5]. Generally, hematite ores containing less than 30% iron are not preferable for extraction from economic point of view until and unless it is not ruled out by some other relevant factors such as superficial location of ores, cheap economy of extraction, and surface located ores. However, dawn of newer technology can support the extraction of ores under minimum cost contrary to the conventional methods of extraction.

It is reported that iron (III) oxide has four polymorphs, namely, alpha, beta, gamma, and zeta-iron oxides with molecular representation, *α*-Fe_2_O_3_ (hematite), *β*-Fe_2_O_3_, ϒ-Fe_2_O_3_ (maghemite), *є*-Fe_2_O_3,_ respectively. Out of them, *α*-Fe_2_O_3_ is antiferromagnetic while the remaining all are ferromagnetic [6, 7]. Magnetite (Fe_3_O_4_) is also ferromagnetic substance. Hematite (Fe_2_O_3_) is a reddish-brown, heavy, and relatively hard oxide mineral of iron. It is harder and more brittle than pure iron. Fe_2_O_3_ possesses a corundum structure specifically trigonal hexagonal scalenohedral, holohedral, and rhombohedral crystal structure, and it is highly stable in ambient conditions [8]. Iron is a major component of hematite ore and forms a principal constituent of steel. The world's iron ore production increased from 274 million tons (in 1950 AD) to 1554 million tons (in 2005 AD) [9]. By the end of the twentieth century, iron consumption for steel manufacture only was standing 850 million tons. It is estimated that the world resource of crude iron ores is approximately 800 billion tones containing more than 230 billion tons of iron [10]. The known resources of iron ores could run out within the next 64 years as the demand for iron is increasing 10% per year. Most of the known deposits contain low-grade ores with iron contents less than 30% [11]. In this context, it is very urgent to search the mines of iron ore, their distribution, deposits, financial, economic, and chemical tests to extract iron from it.

In the case of Nepal, though iron ores were mined and smelted sporadically even before 1951 (2007 BS), no significant progress has been made hitherto [12]. Preliminary studies show some iron deposits in Nepal. Though the occurrences of iron ores are known in many places but only four occurrences in notable quantity are reported. They are Phulchoki iron deposit in Lalitpur district. Those iron deposit in Ramechhap district, Labdi iron deposit in Tanahu district, and Dhaubadi iron deposit in the East Nawalparasi district. The estimated total ore content in these four places is 124.1 million tons [13, 14]. By now, Dhaubadi iron deposit is quantitatively the largest mine of iron reported in Nepal. Dhaubadi area of Nawalparasi district shows 25–100-m thick hematite mineralized bed with a strike length of about 10 km from Pokhari to Dhaubadi at 3–5-km width in preliminary and follow-up exploration [15]. To establish the project for the extraction of iron from Dhaubadi iron deposits, geographical, deposition estimation, economic, and financial estimation have been carried out. It is reported that the return of investment (ROI) is 34% and hence it is an economical and beneficial project for the extraction of a large amount of iron ore from the Pokhari area [16]. The iron ores of Dhauwadi are expected to be extracted more economically due to its superficial location compared to the other known iron reserves. This new reserve is important due to its relatively high deposit of iron ore in addition of being rural area and low population density which supports for the relatively lower cost of investment.

The chemical compositional analysis is also a crucial step to establish an extraction project. Based on the percentage of iron content, financial and economical investment can be estimated. In this context, from the chemist view point, it is utmost necessary to determine the iron content precisely. For this purpose, various chemical techniques and procedures can be adopted. Commonly used methods for the determination of iron content are X-ray fluorescence spectroscopy, atomic absorption spectroscopy, or plasma atomic emission spectrometry [1]. For high iron content sample, titrimetric analysis can be a good method for quantitative analysis. Besides this, other precise methods of quantitative analysis such as determination of concentration of iron by UV-visible spectrophotometric method and atomic absorption spectroscopy method can be adopted. The quantitative analysis of iron can be studied under different parameters like finding the loss on ignition (LOI), sulphur content, acid insoluble matter, and percentage iron content. Based on the findings, the appropriate methods of extraction can be recommended. For instance, iron ores pellets are essential input materials in the manufacture of steel which can be reduced by reducing gas (a mixture of hydrogen and nitrogen gas) [17]. In the work of Jonathan et al., iron was separated from zinc mine tailings via wet magnetic separation followed by carbothermal reduction of self-reducing briquettes [18]. Jorge et al. developed a self-reducing briquettes using biomass source such as charcoal, palm oil charcoal, eucalyptus bark charcoal, and basic oxygen furnace dust [19]. All in all, before embarking the extraction process, it is the parts and parcel to assess the ores chemically.

In this work, it is hypothesized that the presence of some volatile impurities, moisture and acid insoluble impurities in hematite ore, largely affects on the determination of percentage of iron. Therefore, this study aims to find the percentage of extractable iron from the ore besides finding loss on ignition (LOI), sulphur content, and acid insoluble matter in the sample. Furthermore, qualitative and quantitative estimation of iron from the hematite ore is also carried out by UV-Vis analysis and AAS method. Total iron content is determined by titrimetric method as per the protocol of IS: 1493 (part 1): 1981 method [20]. A comparative data of iron content in the hematite ore has been presented in this work.

## 2. Materials and Methods

### 2.1. Materials

Conc. Hydrochloric acid (HCl, Mol. Wt. 36.46, purity 36%, 1.18 g·cm^−3^), conc. Nitric acid (HNO_3_, M. Wt. 63.01, purity 65%, 1.39 g·cm^−3^), conc. Sulphuric acid (H_2_SO_4_, Mol. Wt. 98.08, purity 98%, 1.83 g·cm^−3^), phosphoric acid (H_3_PO_4_, Mol. Wt. 98, purity 85%, 1.88 g·cm^−3^), potassium dichromate (K_2_Cr_2_O_7_, Mol. Wt. 294.19, purity 99.9%, 2.68 g·cm^−3^), diphenylamine sulphonate ((C_6_H_5_)_2_NSO_3_Na, Mol. Wt. 271.26), stannous chloride (SnCl_2_.2H_2_O, Mol. Wt. 225.63, purity 98%), mercuric chloride (HgCl_2_, Mol. Wt. 271.52, 5.43 g·cm^−3^), ammonium iron (III) sulphate dodecahydrate (FeNH_4_ (SO_4_)_2_.12H_2_O, M. Wt. 482.19, Purity ≥98.5%, density 1.71 g·cm^−3^), potassium thiocyanate (KSCN, Mol. Wt. 97.18, 1.89 g·cm^−3^), and hydrogen peroxide (H_2_O_2,_ Mol. Wt. 34.01, assay ≥30.0%, 1.11 g·cm^−3^) were purchased from Merck Life Science, Pvt. Ltd. All reagents were of analytical grades and were used without any further purification. Hematite ores collected from *Pokhari, Nawalparasi* were used for analysis.

### 2.2. Sample Collection

**Figure 1:**
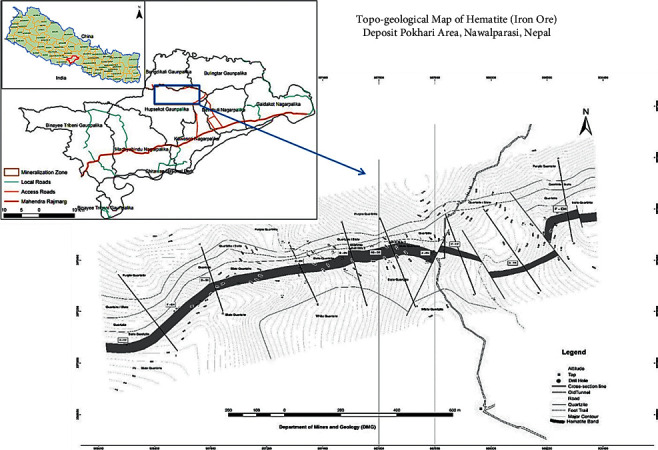
Topo-geological map of the sample collection site Pokhari, Nawalparasi, Nepal [21].

Seventy-two hematite samples were collected from the Pokhari area of the east *Nawalparasi* district by channel sampling method. All samples were collected from 8 different channels, each channel containing nine samples. Channels were drilled 750 m apart. The topo-geological map of the sample collection site is given in Figure 1.

### 2.3. Determination of Loss on Ignition

Each sample was converted into chips with the help of a grinding machine then pulverized into fine powders of about 200 mesh (74 microns). About 20 gram of each sample was dried in an oven at a temperature of about 100°C for an hour and allowed to cool down inside a desiccator. A known weight (1 g) of each sample was taken in a crucible and allowed to heat at a temperature of 1000°C for one hour in an electric muffle furnace (NABERTHERM, LE6/11/R7, Germany) at a rate of 10°C/min to expel all volatile impurities and to oxidize all iron content into iron oxide. Then the sample in crucible was cooled in controlled condition. Then, weight of each sample was measured using 4-digit balance and weight difference before and after ignition was calculated [22, 23].

### 2.4. Gravimetric Determination of Sulphur Content

The iron ore is likely to be associated with sulphide (Iron pyrite, chalcopyrite and others) and sulphate ores for which sulphur estimation is inevitable [24]. In this work, sulphur content was determined gravimetrically. All the sulphur (sulphate sulphur) was targeted to convert into barium sulphate. Any sulphide ions can be precipitated by treating with barium chloride but this work was limited for the estimation of sulphate sulphur [25]. For the determination of sulphur in the sample, 0.5 g of dry ore sample was taken into a 500 mL beaker, 70 mL of acid mixture (60 mL HCl and 10 mL HNO_3_) was added, and then heated at 110°C to evaporate till dryness followed by cooling. Then, 30 mL of dil. HCl was added in it, warmed, 25 mL of water was added on it, and then boiled for 5 minutes. The solution was filtered through Whatman no. 40, and the filtrate was collected into a beaker and evaporated to 25 mL and cooled. 3 mL (1 : 1) HCl and 50 mL water were added in it and then heated. Then, 10 mL of 10% BaCl_2_ solution was added on hot solution slowly and the solution was heated to the boiling for 5 minutes. The white precipitate of barium sulphate was filtered off through Whatman no. 42 and washed to make it free from impurities. The precipitate was dried and ignited into an electric muffle furnace at 500°C for 30 minutes then 800°C for another 30 minutes until a constant mass was obtained, and the amount of sulphur was calculated.

(1) $$\begin{array}{c} \%of\text{ Sulfur}=\left\{\frac{\left(m_{1}-m_{2}\right)\times 0.1374}{m_{3}}\right\}\times 100, \end{array}$$

where *m*_1_ is the mass in grams of platinum crucible and BaSO_4_, *m*_2_ is the mass in grams of platinum crucible only, and *m*_3_ is the mass of sample taken.

### 2.5. Determination of Acid Insoluble Matter (AIM)

About 2.5 gram of each dried sample was taken into a 300-mL capacity beaker and the samples were digested with 35 mL of conc. HCl and 10 drops of conc. HNO_3_ putting on a hot plate at about 100°C temperature. When samples were dried, it was further heated for half an hour. The samples were leached with 10 mL of conc. HCl and then added 20 mL of water. The solution was filtered through Whatman no. 40 filter paper. The filtrate was collected into a volumetric flask and the volume was made 500 mL. The acid-insoluble residue was heated into a muffle furnace for an hour at a temperature of 1000°C, and then the percentage of acid insoluble matter or silica was calculated [22].

### 2.6. Titrimetric Determination of Iron Content

In this experiment, the total iron content was measured by the titrimetric method. Total iron content in the sample was determined as per the protocol of IS: 1493 (part 1): 1981 method. For this, 20 mL of aliquot was titrated against 0.1-N potassium dichromate solution using diphenylamine sulphonate as an indicator in the presence of stannous chloride, mercuric chloride, a mixture of H_2_SO_4_-H_3_PO_4_ reagents. Here, sulphuric acid-phosphoric acid combination was used for leaching. In a similar work, AMilton et al. also used sulphuric acid-phosphoric acid for leaching [26]. Total iron present in a given ore is calculated by the following formula [23]:

(2) $$\begin{array}{c} \text{Total Fe}\left(\text{in}\%\right)=\left(\frac{55.85\times \text{V}\times \text{N}}{1000\times \text{Wt. of sample taken in g}}\right)\times 100\times \text{dilution factor.} \end{array}$$

Here, V is the volume of aliquot in mL and N is the normality of solution.

### 2.7. UV-Vis Spectrophotometric Determination of Iron Content

It is reported that the iron content in the ore from Europe and Brazil measured by the titrimetric method was highly correlated with spectrophotometric measurement [27]. The amount of iron content present in the ores was measured by UV-Vis spectrophotometric method. For this purpose, 100-ppm standard reference solution was prepared by dissolving ammonium iron (III) sulphate dodecahydrate in deionized water.

#### 2.7.1. Method Validation

Different concentrations of iron (III) solution (80, 40, 20, 10, 2.5, 1, 0.50, 0.25 ppm) were prepared by serial dilution of 100-ppm solution [23]. For color development, 1 mL of 0.1 M KSCN solution was added into 9 mL of each diluted solution. The double beam UV-Vis spectrophotometer (Labtronics, Model LT-2802) was used for the measurement of absorbance of the solution [28]. The method validation is useful to determine the limit of detection (LOD = SD of blank solution × 2), limit of quantification (LOQ = SD of blank solution × 10), linearity range, and measurement of uncertainty (MU). The measurement of uncertainty was evaluated from the relation, MU = SD of any measured solution × 2.

#### 2.7.2. *λ*_max_ Determination

The wavelength for maximum absorption was determined by taking 2.5-ppm standard solution.

#### 2.7.3. Preparation of Calibration Curve

The calibration curve was plotted by taking a series of standard ferric thiocyanate (0.5, 1, 5, 10 ppm) solution. This calibration curve was used to determine the concentration of iron in the analyte solution.

### 2.8. Determination of Iron Content by AAS Method

The quantitative estimation of iron in the hematite ores collected from Pokhari, Nawalparasi District was carried out by Atomic Absorption Spectroscopy (AAS) method (SHIMADZU, Model AA-7000, Japan). In this method, 1 g of powder sample was taken and 10 mL of 1 : 1 HNO_3_ was added and refluxed for 10 minutes, followed by the addition of 5 mL concentrated HNO_3_ and refluxed till the total volume was reduced to 5 mL. In the reduced volume, 2 mL of water was added followed by the addition of 3% of H_2_O_2_ until the bubbling subsides. The solution was further refluxed to reduce volume up to 5 mL. Then, 10 mL of concentrated HCl was added and refluxed for 15 minutes. The whole sample was filtered through Whatmann no. 42. The filtrate was taken and diluted to 100 mL in a volumetric flask [29]. This was taken as an analyte sample.

#### 2.8.1. Method Validation

For the method validation of iron estimation, different concentrations (from 0.25 to 100 ppm) of iron (III) were prepared and the blank solutions were taken. Then their absorbance was measured in AAS.

#### 2.8.2. Preparation of Calibration Curve

Standard Fe (III) solutions of 0.5, 1, 5, 10 ppm concentrations were taken and their absorbance was measured to prepare a calibration curve.

## 3. Results and Discussion

### 3.1. Loss on Ignition

**Figure 2:**
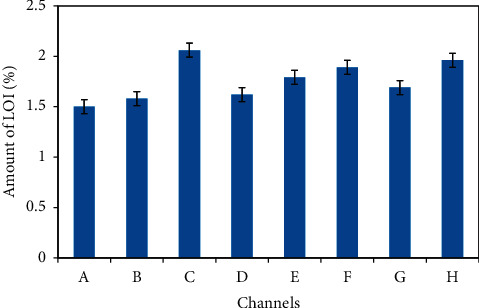
Average percentage of LOI in hematite ore of Pokhari. The presented values are the average of nine samples for each channel ranged from A to H.

Loss on ignition (LOI) is particularly performed at high temperature (in the range of 1000°C) for minerals. It represents organic as well as inorganic carbons, water, and volatile impurities which are chemically bound in the minerals. Strong heating (ignition) of an analyte at a specified temperature expels the volatile substances, organic and inorganic carbons. Metallic ores consist of moisture, water of crystallization, and other associated volatile impurities. The total volatile impurities contain carbonate, moisture, water of crystallization, sulphur dioxide, etc. Moreover, the LOI in hematite ore gives the rough proportion of the carbonate content, but not enough to claim actual content. The data obtained for each channel samples are presented in Table 1. Here, the value of LOI in the measured sample is in the range of 0.75 to 3.75% with mean ± SD value of 1.76 ± 0.17 as presented in Figure 2, indicating good level of precision in the result. This value is less in comparison to those obtained in the analysis of bauxite residue (14.5% and 14.8%, respectively, at 900°C and 1100°C) from a press filter system in the work of AMilton et al. [30]. Therefore, it can be said that the loss on ignition is relatively less in the hematite ores. The loss on ignition of the material reflects the actual materials lost during smelting or refining in a furnace. The weight loss could be attributed to the loss of volatile impurities, hydrates and labile hydroxyl compounds, and carbon dioxide from carbonates.

### 3.2. Gravimetric Determination of Sulphur Content

To find the amount of sulphur content in the hematite ore as an impurity, experiments were carried out but sulphur was not found in measurable quantity.

### 3.3. Determination of Acid Insoluble Matter (AIM)

**Figure 3:**
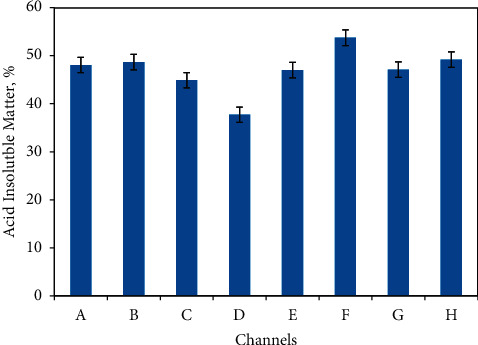
Average percentage of acid insoluble material in hematite ore of Pokhari determined gravimetrically. Samples were collected from eight channels each consisting of nine samples. The values shown here are average of these nine samples of each channel. The channels are arbitrarily graded from A to H.

Silicon dioxide is a principal earthy material associated with iron ores. Therefore, a lower percentage of silicon dioxide reflects a higher percentage of iron. In this work, the acid insoluble matter was estimated based on the gravimetric estimation. The percentage of AIM in every sample is found in between 37 and 54% in the ore as represented in Table 2 and Figure 3. The majority of the acid insoluble material is considered to be silica, an earthy material. This value is a bit higher than a theoretical value. In the similar work on the magnetite-hematite ore of Mikhailovskoye, Nikolaeva reported the average content of silica in the range of 39.0–41.80% [31]. The hematite ores with minimum silica content are easy to remove the silica and the ores are considered to be profitable for the extraction of iron from the economic point of view. Nitric acid reacts with metal and hence oxidizes it. Even though the amount of acid insoluble material is a bit high, the hematite ore of the selected area is still not bad to establish the iron extraction industry.

### 3.4. Titrimetric Determination of Iron Content

**Figure 4:**
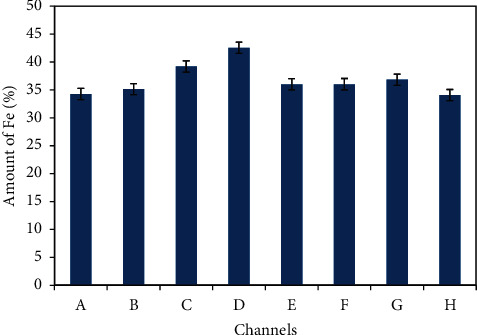
Average percentage of Fe content in hematite ore of Pokhari determined titrimetrically. Samples were collected from eight channels and each channel consists of nine samples. The values shown here are the average of these nine samples of each channel. The channels are arbitrarily graded from A to H.

Iron is the major constituent of hematite ore. The collected samples of hematite ores from all channels were analyzed in the lab by titrimetric method. In this method, sulphuric-phosphoric acid combination is used for leaching, especially alumina [30]. In this work, experimental finding shows that Fe ranges from 34.06 to 42.53%. These values are expressed in Table 3 and in Figure 4. The mean value is 36.75 ± 2.50 in terms of iron percentage. In the similar work, the iron content in magnetite-hematite ore of Mikhailovskoye as reported by Nikolaeva was also found in the range of 38.3–40.1% for which they regarded as “low-Fe grade”. In the work of Jonathan et al. the concentrated materials showed 40.4% of iron where hematite ore was concentrated by flotation process [32]. Our findings of iron content in hematite ore are around the same value as reported by Nikolaeva [31].

Theoretical value of iron in ultra-pure hematite ore is 70% and the ore containing less than 30% iron is not preferable for extraction until and unless the extraction is supported by low cost of extraction and market price of iron. However, the value obtained in this work falls in the low-grade range and prospects to establish an extraction industry. Here, it is to be emphasized that, not only the percentage iron content but also the total amount of deposition of ore determines the fate of iron extraction industry. Iron estimation of hematite ore by titration method, LOI, and SiO_2_ content in the samples assigned from A to H is tabulated in Table 4.

### 3.5. UV Spectrophotometric Determination of Iron Content

Iron solution only does not exhibit any peak in spectrophotometric determination. Therefore, iron (III) was made complex with potassium thiocyanate to develop potassium hexathiocyanatoferrate (III) which can exhibit a color by interacting with photon of UV-Vis energy range. The chemical reaction takes place as follows:

(3) $$\begin{array}{c} {\text{FeCl}}_{3}+6\text{KSCN}⟶{\text{K}}_{3}\left[\text{Fe}{\left(\text{SCN}\right)}_{6}\right]+3\text{KCl} \end{array}$$

**Figure 5:**
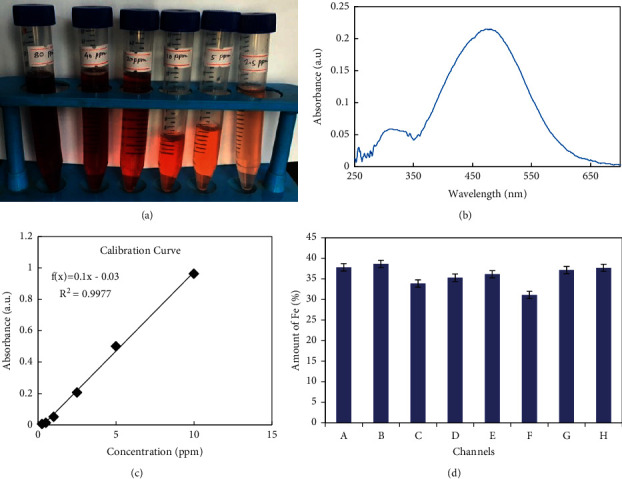
(a) Color development of potassium hexathiocyanatoferrate (III). (b) Determination of *λ*_max_ of the ferric thiocyanate complex using its 2.5-ppm solution. (c) Calibration curve for determination of Fe (III) in the form of Fe (SCN)_3_ by UV spectroscopic method. (d) Amount of Fe in the given ore by UV spectroscopic method.

Method validation was carried out by taking a series of solution having concentrations of 80, 40, 20, 10, 2.5, 1, 0.50, 0.25 ppm as in Figure 5(a). From the measurement of UV-visible spectrophotometer, it was found that the limit of detection (LOD) was found to be 0.25 ppm. The limit of quantification (LOQ) was found to be 2.5 ppm, and the measurement of uncertainty (MU) was determined to be 0.090. The linearity of the curve was found in the range of 2.5 to 20 ppm with an R^2^ value of 0.998. For the measurement of the concentration of analyte solution, *λ*_max_ had to be determined for which 2.5-ppm standard solution was used. A smooth parabolic curve maximum absorbance at 473 nm was obtained as in Figure 5(b). This wavelength was taken as *λ*_max_. The calibration curve with R^2^ = 0.998 was established by taking standard ferric thiocyanate solution as in Figure 5(c). This calibration curve was used to determine the concentration of iron in the analyte solution through which % of the iron in the hematite ore sample was calculated. The amount of iron was determined as in Figure 5(d). The iron content values of different samples of hematite ore determined by UV spectroscopic method are given in Table 5.

### 3.6. Determination of Iron Content by AAS Method

**Figure 6:**
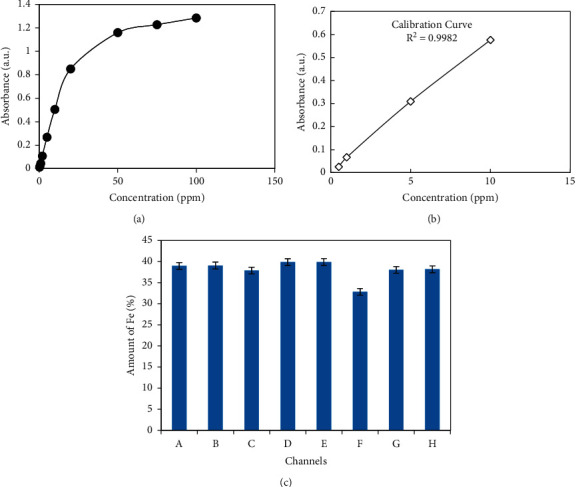
(a) Diagram obtained by plotting absorbance of the different concentrations of standard solutions of Fe (III) for validation. (b) Calibration curve for determination of Fe (III). (c) Amount of iron present in the ore measured by the AAS method.

In the determination of iron by AAS method, the uses of nitric acid react with metal and hence oxidizing it. In some cases, high silica content could be the problem in leaching step as it may cause silica gel formation. The silica gel formation can be avoided using hydrogen peroxide [30]. The standard deviation obtained by measuring the absorbance of the blank solution, limits of detection (LOD), and limits of quantification (LOQ) were calculated and found to be 0.003616 and 0.012054, respectively. Similarly, the linearity range was calculated and found from 0.25 to 20 ppm with R^2^ = 0.998. Measurement uncertainty (MU) was determined by taking the reference of 2-ppm solution and found to be ±0.011. Method validation is further expressed in the following diagram 6(a). Standard Fe (III) solutions of 0.5, 1, 5, 10 ppm concentrations were taken and their absorbance was measured to prepare a calibration curve. The calibration curve obtained for this purpose was given in Figure 6(b) with an R^2^ value of 0.998.

After the preparation of the calibration curve, the amount of iron present in the analyte sample was measured. The percentage of iron content present in the samples was found similar to the titrimetric method. These are shown in Figure 6(c). Iron content values of different samples of hematite ore determined by AAS measurement method are shown in Table 6.

### 3.7. A Comparative Study

**Figure 7:**
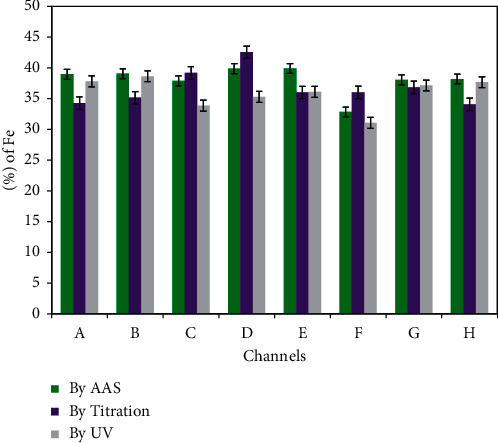
Amount of iron (%) in different samples by three different methods.

A comparative study shows that total eight samples, one from each channel were measured by the AAS method and compared with the UV-Visible Spectroscopic method and titrimetric method. The results obtained by all three methods are presented in Figure 7 and Table 7.

Though each method has its own pros and cons, AAS method is highly preferable and more accurate in comparison to others. It is because the data obtained by this method are highly précised. Taking example of channel *D*, the mean values for iron content measured by AAS, UV, and titration methods are 39.87, 35.28, and 42.53, respectively. Here, the value obtained by titration method is higher than that of AAS method. The reason behind it may be precise and careful visual and volumetric errors in titrimetric analysis. Such errors are not present in the instrumental methods so that they give more accurate results. But, the value obtained by UV measurement is lower than among. This could be more accurate but as we found that UV-Vis has high MU value, this may less precise value in comparison. The value obtained by AAS method is intermediate with high precision and could be more accurate. Also AAS has lower MU and less relative standard deviation in the results obtained by this method. All in all, results obtained from these three methods are comparable.

## 4. Conclusion

Total iron content and other parameters are measured by the titrimetric method using a standard protocol. The determined values show that the total iron content in the sample is more than 36% representing the intermediate content. All in all, the adopted methods of test, i.e., AAS, UV-vis, and titration give the near value of iron content. The result showed a low-iron grade ore. Establishment of industry is influenced by some other factors such as processing cost, labor costs, and market prices. Considering several other factors determining the fate of running an iron industry, operation of iron industry could be feasible in case of high reservation of iron on relatively shallow mining state. As the extension of ore is about 10 km in length, 3–5 km in width, and 100 m deep with the expectation of about 100 million tons of reserved iron, the iron extraction industry could be profitable.

## Figures and Tables

**Table 1:** Loss of ignition (LOI) values of different samples of hematite ore (in percentage). The mean value in the table represents the mean of nine samples for each channel assigned from A to H.

Sample	1	2	3	4	5	6	7	8	9	Mean
A	1.54	0.98	1.02	1.96	2.01	1.76	1.24	1.08	1.92	**1.50**
B	1.62	1.89	2.04	1.60	0.75	1.38	2.50	1.36	1.12	**1.58**
C	1.54	1.72	2.28	1.96	2.01	2.01	2.00	2.08	2.92	**2.06**
D	0.92	1.70	1.48	1.65	2.11	1.61	1.88	1.76	1.46	**1.62**
E	1.72	1.74	1.70	1.35	2.02	1.55	1.74	2.42	1.91	**1.79**
F	1.37	3.13	2.05	1.68	1.16	1.12	3.59	1.23	1.74	**1.89**
G	1.62	1.89	2.04	1.60	3.75	1.38	0.50	1.36	1.10	**1.69**
H	1.64	2.41	1.90	1.85	2.77	1.86	1.79	2.34	1.16	**1.96**

**Table 2:** Acid insoluble material values of different samples of hematite ore (in percentage). The mean value in the table represents the mean of nine samples for each channel assigned from A to H.

SN	1	2	3	4	5	6	7	8	9	Mean
A	44.24	49.45	51.86	49.05	50.92	42.13	49.20	47.31	48.48	**48.07**
B	44.38	43.64	51.42	42.99	55.97	48.61	48.36	46.47	56.31	**48.68**
C	49.61	57.41	44.05	47.76	46.69	47.72	37.45	40.58	33.04	**44.92**
D	36.08	36.47	31.55	43.71	35.70	41.87	35.43	33.13	45.64	**37.73**
E	43.08	42.92	42.53	50.03	58.71	38.91	45.69	48.82	52.35	**47.00**
F	67.82	46.90	52.12	55.4	62.66	46.65	61.26	44.74	46.44	**53.77**
G	63.51	47.30	43.88	46.34	41.76	37.28	46.55	50.63	46.82	**47.11**
H	51.38	46.75	48.67	52.19	53.12	46.31	47.61	52.23	44.52	**49.19**

**Table 3:** Iron content values of different samples of hematite ore. The mean value in the table represents the mean of nine samples for each channel assigned from A to H.

Sample	1	2	3	4	5	6	7	8	9	Mean
A	37.02	34.06	32.5	32.89	32.81	37.89	33.86	34.04	33.29	34.26
B	36.85	36.26	36.84	35.17	32.7	34.62	34.6	35.7	33.49	35.14
C	36.41	36.2	40.5	40.12	39.2	39.1	40.13	41	40	39.18
D	42	39	46.64	38.5	43.89	42.61	44.96	46.47	38.7	42.53
E	37.87	37.92	38.58	33.51	32.33	40.73	36.28	34.05	32.83	36.01
F	31.26	36.27	35.17	32.71	34.8	41.32	33.94	39.44	39.33	36.02
G	34.04	40.53	35.79	35.08	38.47	39.54	36.05	33.38	38.52	36.82
H	32.81	34.02	33.65	33.74	32.26	33.98	38.47	33.64	34.02	34.06

**Table 4:** A comparative study of LOI, SiO_2_, and Fe estimation of hematite ore by titration method. The mean value in the table represents the mean of each channel samples assigned from A to H.

Sample	LOI	SiO_2_	Fe
A	1.50	48.07	34.26
B	1.58	48.68	35.14
C	2.06	44.92	39.18
D	1.62	37.73	42.53
E	1.79	47.00	36.01
F	1.89	53.77	36.02
G	1.69	47.12	36.82
H	1.96	49.19	34.06
Mean	1.76	47.06	36.75
SD	0.17	4.01	2.83
Mean ± SD	1.76 ± 0.17	47.06 ± 4.01	36.75 ± 2.83
Relative SD in percentage	9.6	8.52	7.7

**Table 5:** Iron content values of different samples of hematite ore determined by the measurement of absorption using spectrophotometer. The mean value in the table represents the mean of samples for each channel assigned from A to H.

Sample	Absorbance	mg/kg	% Fe	Mean Fe	SD	Mean ± SD	% of SD
A	1.161	378093.7087	37.8093	35.94	2.50	35.94 ± 2.50	6.9
B	1.164	386315.3831	38.6315				
C	1.132	338617.4375	33.8617				
D	1.142	352528.7932	35.2528				
E	1.158	361086.9144	36.1086				
F	1.069	310591.7532	31.0591				
G	1.158	371364.0923	37.1364				
H	1.159	376765.2251	37.6765				

**Table 6:** Iron content values of different samples of hematite ore determined by the AAS measurement method. The mean value in the table represents the mean of samples for each channel assigned from A to H.

Sample	Absorbance	mg/kg	% Fe	Mean	SD	Mean ± SD	% of SD
A	0.9575	389564	38.9564	38.09	2.26	38.09 ± 2.26	5.93
B	0.9684	390482	39.0482				
C	0.8897	378872.8	37.88728				
D	1.0489	398702.7	39.87027				
E	0.9978	399112	39.9112				
F	0.7879	328200.8	32.82008				
G	0.9535	380381.6	38.03816				
H	0.9398	381667.3	38.16673				

**Table 7:** A comparison of iron content present in hematite ore by AAS, titration, and UV-spectrophotometric method.

Channels	Percentage of Fe determination		
	By AAS	By UV	By titration
A	38.96	37.80	34.26
B	39.05	38.63	35.14
C	37.89	33.86	39.18
D	39.87	35.28	42.53
E	39.91	36.11	36.01
F	32.82	31.05	36.02
G	38.04	37.13	36.82
H	38.17	37.67	34.06
Average	38.09	35.94	36.75
SD	2.26	2.50	2.83
Average ± SD	38.09 ± 2.26	35.94 ± 2.50	36.75 ± 2.83
Relative SD in percentage	5.93	6.95	7.70

## Data Availability

The data used to support the findings of this study are available from the corresponding author upon request.

## Additional Points

*Highlights.* Seventy-two samples of hematite ores were collected from Nawalparasi. LOI, sulphur content, and acid insoluble contents in the samples were studied. Titrimetric, UV analysis, and AAS methods were applied for the analysis of Fe. Collected samples have intermediate Fe content.
